# Early-life stages impact later feeding behavior and physiology in light-born piglets

**DOI:** 10.1093/jas/skaf437

**Published:** 2025-12-18

**Authors:** Elizabeth Huenul, Susana M Martín-Orúe, Lluís Fabà, Ruth Forsten, Pau Salgado-López, Ferran Llobet-Cabau, María José Rodríguez-Lagunas, Marta Fornós, José Francisco Pérez

**Affiliations:** Animal Nutrition and Welfare Service (SNiBA), Department of Animal and Food Science, Autonomous University of Barcelona, Bellaterra 08193, Spain; Animal Nutrition and Welfare Service (SNiBA), Department of Animal and Food Science, Autonomous University of Barcelona, Bellaterra 08193, Spain; Trouw Nutrition R&D, Swine Research Centre, Boxmeer, JN 5831, The Netherlands; Physiology Section, Department of Biochemistry and Physiology, Faculty of Pharmacy and Food Science, University of Barcelona, Barcelona, Spain; Animal Nutrition and Welfare Service (SNiBA), Department of Animal and Food Science, Autonomous University of Barcelona, Bellaterra 08193, Spain; Plant and Animal Genomics, Centre for Research in Agricultural Genomics (CRAG), CSIC-IRTA-UAB-UB Consortium, Bellaterra 08193, Spain; Physiology Section, Department of Biochemistry and Physiology, Faculty of Pharmacy and Food Science, University of Barcelona, Barcelona, Spain; Animal Nutrition and Welfare Service (SNiBA), Department of Animal and Food Science, Autonomous University of Barcelona, Bellaterra 08193, Spain; Animal Nutrition and Welfare Service (SNiBA), Department of Animal and Food Science, Autonomous University of Barcelona, Bellaterra 08193, Spain

**Keywords:** depression, feeding behavior, growth, nursery, physiology, suckling

## Abstract

Low birth weight (LBW) piglets and those exposed to negative experiences early in life are at higher risk of poor development. This study aimed to investigate the potential imprinting effects of early growth rates on metabolism and feeding behavior in LBW piglets. A total of 128 piglets ([*Landrace × Yorkshire*] *× Pietrain*) were selected at weaning 23.1 ± 0.37 d old (5.2 ± 0.94 kg body weight [BW]), including 69 castrated males and 59 females. At 51 d of age, 46 LBW piglets (0.98 ± 0.139 kg) were classified using a 2 × 2 factorial design based on growth rate during the suckling and nursery periods. Average daily gains for fast- and slow-nursery piglets were 315 ± 36.6 and 230 ± 39.0 g/d in the fast-suckling group, and 236 ± 26.9 and 159 ± 21.1 g/d in the slow-suckling group. Thereafter, piglets were euthanized at 58 d-old for sampling of physiological biomarkers in blood and urine, and jejunal gene expression. Postweaning piglet to feeder interactions were recorded daily and BW was measured weekly. Slow-growth piglets during the suckling and nursery periods spent less time at the feeder per day during the first week post-weaning (P < 0.05). Additionally, slow-growing suckling piglets showed a tendency to higher urea (*P* = 0.087) and lower albumin (*P* = 0.079) levels, and significantly lower Zn and Gamma-Aminobutyric Acid (*P* < 0.05) concentrations in blood compared to fast-growth piglets. In urine, the suckling slow-growing piglets had significantly higher urea, sulfur, kynurenine and tryptophan creatinine ratios (*P* < 0.05), with a trend toward a higher kynurenine/tryptophan ratio (KTR; *P* = 0.084). Slow-growing nursery piglets presented a low level of Zn in blood, a high level of KYN/Creatinine (*P* < 0.05) and tended to a high KTR (*P* = 0.074) in urine. Regarding jejunal gene expression, slow-growing suckling piglets showed a significant higher mRNA abundance of the immune response gene *CXCL2* (*P* = 0.043) with a tendency toward increased mRNA levels of *S100A9* and *IL-8*. Furthermore, there was a tendency to higher mRNA abundance of the protein breakdown gene (*SH3RF2*) and stress response gene (*HSD11β1*) (*P* < 0.10). The results suggest that the effects of the suckling and nursery periods were often independent and early-life experiences in LBW piglets, especially the suckling period, have a significant impact on their overall performance, as reflected in the physiological and behavioral differences observed at the end of the nursery period.

## Teaser

Early-life experiences, especially during the suckling period, significantly influence piglets’ growth, physiology, and behavior by the end of the nursery period.

## Introduction

The drive for maximal productive efficiency in intensive pig production has led genetic selection to focus on increasing litter size through the selection of hyper-prolific sows. However, this has resulted in 20% to 27% of piglets being born with low birth weight (LBW; <1.2 kg), a 20% to 25% increase in body weight (BW) variability, a 40 g reduction in average daily gain (ADG), and only a 58% survival probability until weaning (Quiniou et al., 2002; López-Vergé et al., 2018). This reduces batch uniformity, mainly due to low-growth piglets exhibiting limited growth potential, reduced metabolic efficiency, and impaired immune function (He et al., 2016). These factors may contribute to prolonged time to reach slaughter weight, increased susceptibility to disease, mortality rates, and BW variability (Wolter and Ellis, 2001; Douglas et al., 2014; López-Vergé et al., 2018; Camp Montoro et al., 2020). Consequently, such outcomes negatively affect animal welfare, farm sanitary status, and production efficiency, leading to economic losses for the producer (Calderón Díaz et al., 2017).

Early piglet development, as well as events occurring during the suckling and nursery periods, is thought to exert an imprinting effect that is critical and directly impacts the future performance of the animals. During the suckling period, colostrum and milk provide passive immunity, growth factors, peptides, immuno-active molecules, and nutrients to support the development of piglets (Picone et al., 2018), which may influence their subsequent physiological responses. Body weight at birth may contribute to inadequate colostrum intake; however, malnutrition or adverse neonatal conditions during the early postnatal period may independently lead to differences in feed efficiency in both the suckling and post-weaning periods. These factors may also delay myofiber maturation of the piglets, thereby potentially affecting carcass quality (Romero et al., 2022).

During the nursery period, management strategies aim to promote recovery from weaning stress and improve BW at the start of the fattening period (Lallès et al., 2007). The most important challenge is the transition from mother’s milk to a dry, plant-based diet, as it causes intestinal changes and anorexia. In this regard, Fabà et al. (2024) recently observed that piglets exhibiting an early response to feed intake after weaning, characterized by more frequent and longer visits to the feeder, showed high growth performance. In addition growing pigs, differing in performance exhibited distinct patterns of feeding behaviors (Carcò et al., 2018). However, this information has been obtained using individual electronic feeders that require animal training, particularly during the nursery period, when feeders typically have three to five spaces.

Slow-growing piglets have been associated with lower feed efficiency, in which genes are linked to diverse biological processes, including energy metabolism, immunity, nervous system function, and behavior (Calderón Díaz et al., 2017; Reyer et al., 2017). Whether as a cause or consequence, growing piglets with different growth rates also differed in physiological variables such as length and weight of the small intestine, Insulin-like Growth Factor 1 (IGF-1), insulin, leptin, and amino acid concentrations (Paredes et al., 2014; He et al., 2016). In line with these findings, González-Solé et al. (2022) recently observed that piglets with different growth rates during the suckling period exhibit distinct microbiota composition, fermentation capacity, and glucocorticoid levels by the end of the nursery period.

A previous study has shown that management and nutrition during the nursery period have a similar effect on the performance of all animals (Zeng et al., 2019). However, no studies have investigated whether growth during the suckling and nursery periods are interdependent processes or whether they influence metabolic status and feeding behavior independently of each other, particularly in high-risk piglets with LBW. Therefore, we hypothesized that LBW piglets differing in ADG (Fast vs. Slow) during the suckling and/or nursery period would differ in metabolic and feeding behavior responses at the end of the nursery period, either independently or through interaction. This study aimed to investigate potential early imprinting effects on feeding behavior and metabolism at the end of the nursery period by analyzing piglets grouped according to differences in ADG (Fast vs. Slow) during the suckling and nursery period.

## Materials and Methods

### Ethical approval

Experiments were conducted at the swine experimental facility belonging to the Autonomous University of Barcelona (UAB) in Barcelona, Spain. All experimental procedures were approved by the Ethical Committee on Animal Experimentation of CEAAH 6644 and are in compliance with national legislation following the EU-Directive 2010/63/EU for the protection of animals used for scientific purposes.

### Animals, housing and experimental design

A total of 128 castrated male (*n* = 69) and female (*n* = 59) piglets ([*Landrace × Yorkshire*] × *Pietrain*) were selected from a commercial farm for the study. Piglets from two farrowing batches originating from consecutive weekly sow groups were weighed at birth, on day 7, and at weaning. Piglets were randomly selected from each batch and were transported to the swine experimental facility at the UAB in two consecutive weeks (64 piglets per week) at 23 d old (5.21 ± 0.944 kg). Animals were individually identified by using numbered and chip plastic ear tags (RFID, MS Tag Round HDX STF-YELOPPRPB1, MS Schippers, Spain), weighed, and distributed into 16 nursery pens (1.95 m × 0.95 m; fully slatted floor) in groups of eight piglets per pen. Allocation was based on weaning BW, and piglets were housed in mixed-sex groups. Room lighting was on between 07:00 h and 20:00 h throughout the study and the room temperature was initially set to 29 °C at weaning and gradually (1 °C per week) reduced to 25 °C and maintained until the end of the nursery. Each pen had two individual nipple drinkers and one electronic feeder (TR5 feeder, Rotecna, Spain; Mpigdata S.L. Madrid, Spain) with five feeding spaces. Piglets were given *ad-libitum* access to water and fed a standard commercial diet (pre-starter for three initial weeks and starter for the two following weeks) according to their nutritional requirements (NRC, 2012). A hessian sack hanging from the walls of the pen was used as an environmental enrichment. Animals were weighed individually once a week to obtain the ADG. Their health was monitored daily at the pen level, and they were raised under conditions simulating a commercial farm. At 51 d-old, 46 animals (27 males and 19 females) were selected from the total population of the experimental farm (Table 1), based on their ADG during the suckling and nursery phases. Selection followed a 2 × 2 approach: fast and slow growth groups during suckling period (218 ± 23.4 and 139 ± 17.8 g/d, respectively). During the nursery period, fast and slow growth categories were defined within each suckling group: 315 ± 36.6 vs. 230 ± 39.0 g/d in the fast-suckling group, and 236 ± 26.9 vs. 159 ± 21.1 g/d in the slow-suckling group. A LBW (0.98 ± 0.139 kg) was considered as a secondary criterion. The selected animals were euthanized on day 58 of life for physiological sampling.

**Table 1.** Mean average daily gain (ADG) and body weight (BW) of all animals in the experiment and the subset selected for the study

Fixed effect			Suckling × Nursery				Suckling		Nursery		RSD^2^	*P-*value		
	Groups	*Entire group^3^*	Fast		Slow		Fast	Slow	Fast	Slow		Suckling	Nursery	Suckling × Nursery
			Fast	Slow	Fast	Slow								
***n***	Group size^1^	*128*	12	12	11	11	24	22	23	23				
**Average daily gain, g/d**	Suckling*	*175*	220	214	141	137	217	139	181	176	12.2	<0.001	0.228	0.735
	Nursery*	*242*	315	227	234	163	271	198	275	195	27.5	<0.001	<0.001	0.349
	Day 23 to 30	*215*	277	183	234	162	230	198	255	173	40.9	0.053	<0.001	0.449
	Day 51 to 58	*336*	422	350	273	225	386	249	348	288	74.1	<0.001	0.019	0.635
	Overall	*216*	278	221	197	153	250	175	238	187	18.1	<0.001	<0.001	0.294
**Body weight, Kg**	Birth	*1.25*	1.05	1.02	0.97	0.90	1.03	0.94	1.01	0.96	0.123	0.021	0.245	0.606
	Weaned	*5.21*	6.15	5.99	4.19	4.06	6.07	4.12	5.17	5.02	0.268	<0.001	0.147	0.898
	58-d old	*13.71*	17.2	13.9	12.4	9.8	15.5	11.1	14.8	11.8	1.06	<0.001	<0.001	0.311

1 Number of animals on each group.

2 Residual Standard Deviation.

3 Mean value for all animals used in the experiment and timepoint.

4 Average Daily Gain during suckling and nursery period, from birth to the end of nursery

* Animal selection criteria

Data presented as EMMs.

### Feeding interaction

**Figure 1.**
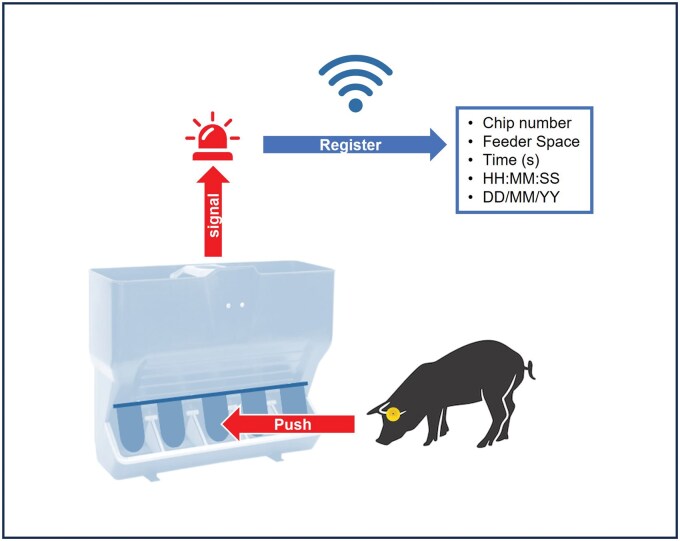
Schematic representation of the electronic feeder function. When the piglet entered a feeder space, it activated a lever with its snout. This action triggered a signal to the feeder circuit, which, via a Wi-Fi antenna, recorded the following data: the animal’s chip number, feeder space, date and time of entry, and duration of the visit. System developed by Mpigdata S.L. Madrid, Spain. Pig: Freepik; feeder: Rotecna. Pig: Freepik (https://www.freepik.es/fotos-vectores-gratis/cerdos-silueta); feeder: Rotecna (https://www.rotecna.com/productos/tolvas/).

The individual feeding behavior of the animals was measured throughout the nursery period using chip plastic ear tags in piglets and an electronic feeder that continuously transmitted wirelessly to a server accessible via a logging system. The feeder recorded the chip number and the time whenever a piglet pushed a lever positioned in each feeding space (Figure 1). With this mechanism, the number of visits to the feeder per day (V/d; frequency), the time spent in the feeder per visit (s/V; seconds), and the time spent in the feeder per day (min/d; minutes) were measured in each pig. Visits that lasted less than 5 s were not considered for the analysis, and a change in feeder space was considered a new visit.

### Sample collection

Selected piglets were sedated with a combination of Zoletil (250 mg zolazepam and 250 mg tiletamine; VIRBAC, Carros, France) and 20 mL Sedanum (20 mg xylazine/mL; Dechra Pharmaceuticals, Northwich, United Kingdom) at 1 mL per 10 kg BW. Subsequently, a 10 mL blood sample was collected from the jugular vein in 10 mL tubes (BD Vacutainer PET Tube, Becton, Dickinson and Company, EE. UU and BD EDTA K2 Tube, Becton, Dickinson and Company, EE. UU) and then animals were humanely euthanized via jugular injection with 40% barbiturate pentobarbital (390 mg pentobarbital sodium and 50 mg phenytoin sodium per mL, Euthasol, Virbac, Carros, France) at a dose of 0.5 mL/kg -BW. at 58 d-old. Blood was maintained on ice for up to four hours until serum and plasma were obtained by centrifuging at 2,000 g for 10 min at 4 °C and stored in 2 mL Eppendorf tubes (Eppendorf Safe-Lock Tubes, Eppendorf, Alemania) at −20 °C until further analysis. After euthanasia, urine and jejunum tissue samples were collected. A total of 6 mL of urine was collected by bladder puncture using a sterilized 10 mL syringe (Sterile 10 mL syringe, Deltalab, Spain) with a 20G needle, transferred to 2 mL Eppendorf tubes and stored at −80 °C for further analyses. Abdominal organs were obtained and the distal jejunum was identified as the tissue located approximately ∼3 m proximal to the ileocecal junction, from which a 0.5 cm^2^ full thickness section was rapidly excised by transverse transection, rinsed in phosphate-buffered saline (PBS), immediately snap frozen in RNAlater (Deltalab, Rubí, Spain) and stored in 2 mL cryotubes (RNase-free sterile cryovials, Deltalab, Spain) at −80 ◦C until further analysis.

### Blood and urine metabolites analyses

Different metabolites were analyzed in blood and/or urine as biomarkers to assess nutritional status (Albumin and Zn in blood), protein catabolism (urea in blood and urine and sulfur in urine), or animal behavior (γ-Aminobutyric acid [GABA] in blood and Kynurenine [KYN] and Tryptophan [TRP] in urine). Creatinine was used to standardize urinary measurements and correct for variability in urine dilution, and each urinary metabolite was expressed as its corresponding metabolite-to-creatinine ratio (Saude et al., 2007). Metabolites were measured using different commercial laboratory protocols. Serum Zinc was determined by Inductively Coupled Plasma Mass Spectrometry (ICP-MS), Model 7900 (Agilent Technologies, United States). Serum albumin was assessed by Bromocresol Green Method, AU480 analyzer (Beckman Coulter, Germany). Urine sulfur was analyzed using an Agilent inductively assembled plasma optical emission spectrometer (ICP-OES), model 5900 (Agilent Technologies, United States); Urine creatinine by Jaffé method, AU480 analyzer (Beckman Coulter, Germany). Urea in urine and blood was measured by the Urease method, kinetic, UV reading AU480 analyzer (Beckman Coulter, Germany). Serum GABA, urine KYN and TRP were measured by High-performance liquid Chromatography (HPLC-MS) model 1260 and Mass Spectrometry (MS) triple quadrupole model 6420 (Agilent Technologies, United States). Finally, plasma cytokines including Interleukin-12 subunit p40 (IL-12p40), Tumor necrosis factor alpha (TNF-α), and Interferon-alpha (IFN-α) were measured using the ThermoFisher Scientific ProcartaPlex Pig Cytokine & Chemokine Panel 9 plex Kit (Luminex model MAGPIX).

### Gene expression

A sample of 30 mg of frozen jejunum tissue was homogenized using Lysing Matrix D 2 mL tubes (MP Biomedicals, Irvine, CA, United States) in a Precellys Evolution tissue homogenizer (Bertin Technologies, Montigny-le-Bretonneux, IDF, FR) coupled with a Cryolys Evolution cooling system (Bertin Technologies, Montigny-le-Bretonneux, IDF, FR) using an homogenization cycle of 6,500 rpm during 30 s at 4 °C. Total RNA was obtained using the Maxwell RSC simplyRNA Tissue Kit (Promega Corporation, Madison, WI, United States) in a Maxwell RSC instrument (Promega Corporation, Madison, WI, United States) following the manufacturer’s instructions. The quantity and purity of RNA were assessed with a NanoDropND-1000 spectrophotometer (NanoDrop products, Wilmington, DE, United States) whereas RNA integrity was assessed via an Agilent Bioanalyzer-2100 equipment (Agilent Technologies, Santa Clara, CA, United States). All samples showed RNA integrity value (RIN) higher than 7. Reverse transcription of approximately 1 µg of total RNA to double-stranded cDNA using random primers was performed using a High-Capacity cDNA Reverse Transcription kit (Applied Biosystems, Foster City, CA) in a reaction volume of 20 μL. The thermal cycler conditions applied were 25 °C for 10 min, 37 °C for 120 min; 85 °C for 5 min, and 4 °C hold.

Jejunal gene expression was analyzed by high-throughput real-time qPCR using a 96.96 Dynamic Array IFC for Gene Expression (Standard Biotools Inc., South San Francisco, CA, United States) in a Biomark HD (Standard Biotools Inc., South San Francisco, CA, United States) system. A cDNA pool representative of all the experimental conditions was included to check real-time qPCR efficiency of each of the analyzed genes, using the relative standard curve method. Triplicates of 2-fold (1/5, 1/10, 1/20, 1/40, 1/80, 1/160), and 4-fold (1/320, 1/1280, 1/5120) serial dilutions were included. Samples were processed per duplicate at 1/20 dilution for the quantification of 45 selected genes (43 target genes and 2 reference genes) based on previous results (Villagómez-Estrada et al., 2022).The genes were selected based on literature involved in intestinal health and grouped according to their main physiological function: 1) barrier function genes such as mucins (*MUC2*, and *MUC13*), zonula occludens (*ZO1*), trefoil factor 3 (*TFF3*), and occludin (*OCLN*); 2) the immune and inflammatory functions such as pattern recognition receptors, cytokines, chemokines, and stress proteins: toll-like receptor (*TLR2, TLR4*), TLR adaptor Myeloid Differentiation Primary Response Protein (*MyD88*), Nuclear Factor Kappa-betta (*NFK-β*), S100 Calcium (and zinc) Binding Protein A9 (*S100A9*), interleukin (*IL1β, IL6, IL8, IL10*), interferon gamma receptor (*IFNGR1*), tumor necrosis factor alpha (*TNF-α*), transforming growth factor beta 1 (*TGF*-*β1*), chemokine ligand (*CXCL2*), heat shock protein (*HSPB1* and *HSPA4*), peroxisome proliferator activated receptor alpha (*PPARGC1α*), fatty acid hydroxylase domain containing 2 (*FAXDC2*), and guanylate binding protein (*GBP1*); 3) antioxidant enzymes genes such as glutathione peroxidase (*GPX2*), superoxide dismutase (*SOD2*); 4) digestive enzyme and hormone genes involved in the digestion and metabolism processes: intestinal alkaline phosphatase (*ALPi*), sucrase-isomaltase (*SI*), d-amino-acid oxidase (DAO), histamine N-methyltransferase (*HNMT*), alanyl aminopeptidase membrane (*ANPEP*), indoleamine 2,3-dioxygenase (*IDO1*), cholecystokinin (*CCK*), IGF-I receptor (*IGF1R*), and E3 Ubiquitin-Protein Ligase (*SH3RF2*); 5) nutrient transport coding genes: solute carrier family (*SLC5A1, SLC7A8, SLC13A1, SLC15A1, SLC16A1*, and *SLC39A4*), Proline-Rich *AKT1* Substrate 1 (*AKT1S1*); and 6) stress response genes: corticotropin releasing hormone receptor 1 (*CRHR1*), and hydroxysteroid (11-beta) dehydrogenase 1 (*HSD11β1*).

Data collection and quality control were performed in the software Fluidigm Real-Time PCR Analysis (Standard Biotools Inc., South San Francisco, CA, United States) (v4.8.1). The software DAG Expression (v1.0.5.6) (Ballester et al., 2013) was utilized for jejunal gene expression analysis. The relative standard curve method was applied to account for the gene-specific efficiency of the qPCR reaction, obtained from the serial dilution of the representative cDNA pool, generating quantity (Q) values. In addition, normalization of these jejunal mRNA abundance was performed using the geometric mean of two reference genes (*GAPDH* and *TBP*), generating the normalized quantity (NQ) relative gene expression values.

### Statistical analysis

Data were analyzed with ANOVA by using linear mixed models with the software R v4.2.2 (R Foundation for Statistical Computing, Vienna, Austria). For the performance the model included suckling and nursery growth as fixed effects, along with their interaction; pen, sow, sex and week of arrival at the facilities were included as random effects. For the physiological variables (blood, urine and gene expression), the model included suckling and nursery growth as fixed effects, along with their interaction. Sex and week of arrival at the facilities were included as random effects, pen and sows had no significant differences in the model therefore were not included. For feeding behavior, a similar model was used; additionally, pen and individual animal were included as random effects to account for the repeated measure’s structure. Before ANOVA analysis, normality and homoscedasticity were checked with the Shapiro–Wilk test and Levene’s test, respectively, for each variable. Outlier values were identified as mean ± 3 SD and removed from the dataset. A Box–Cox transformation was performed on data that did not follow a normal distribution. All results were presented as estimated marginal means (EMMs) adjusted by Tukey and Residual Standard Deviation of the model (RSD). Cohen’s *d* and its CI were reported when trends were observed. Additionally, for gene expression analysis, the false discovery rate (FDR) was applied. The associations between all variables were assessed using Pearson’s correlation analysis and visualized with the ‘corrplot’ package in R (version 4.2.2). To assess the association between variables and immune, digestive, barrier, protein breakdown, antioxidants, and stress function, seven gene expression markers and cytokines that did not differ significantly in the fixed-effect model were included in the correlation analysis. The experimental unit was the piglet in all analyses. Differences at *P* < 0.05 were considered statistically significant and differences at 0.05 ≤ *P* < 0.10 were considered a tendency.

## Results

### Growth performance

The selected piglets in the slow or fast growth classes in the pre and postweaning 2 × 2 exhibited extreme ADG which resulted in around 29% above and below the study mean ADG from birth to day 58 of life (216 g/d) (Table 1). No interaction between suckling and nursery growth rate was observed for any of the performance variables, therefore, the *P*-values reported below correspond to the main effects. Piglets with faster growth during the suckling period had significantly higher ADG during suckling, nursery and overall period (*P* < 0.001), as well as a tendency during the first (23–30 d-old; *P* = 0.053) and significant difference in the last weeks of the nursery period (51–58 d-old; *P* < 0.001). Moreover, these piglets showed significantly higher birth (*P* = 0.021), weaning (*P* < 0.001), and final BW (*P* < 0.001) compared to the slower-growing piglets during the suckling period. In contrast, piglets with faster growth during the nursery period, had significantly higher ADG during nursery, overall period, and both the first and last weeks of the nursery period, as well as 58 d-old BW (*P* < 0.001) compared to the slower growing piglets during the nursery period.

### Feeding behavior

During the first week after weaning no significant difference or tendencies were found in the interaction between suckling and nursery growth rate. Accordingly, the *P*-values reported correspond to the main effects. Piglets with fast growth during suckling period spent more time at day in the feeder (*P* = 0.038) and showed a tendency to make more visits per day (*P* = 0.065) and spend more time per visit (*P* = 0.079) compared to their counterparts with slower growth rates during suckling period. In addition, the piglets that grew fast in the nursery period exhibited more visits per day (*P* = 0.011), similar time per visit (*P* = 0.780) and spent more time per day in the feeder (*P* = 0.016) than the slow-growing pigs. Data are summarized in Table 2.

**Table 2.** Events of visits per day, time per visit and cumulative time per day for the different growth groups during suckling and nursery periods during the first week of nursery (23- to 30-d-old pigs) and in the last week of nursery (51- to 58-d-old pigs)

Fixed effect		Suckling × Nursery				Suckling		Nursery		RSD^1^	*P-*value		
		Fast		Slow		Fast	Slow	Fast	Slow		Suckling	Nursery	Suckling × Nursery
Variables	Units	Fast	Slow	Fast	Slow								
**First week of nursery**													
**Visit/Day**	V/d	90.0	67.6	72.8	37.6	78.8	55.2	81.4	52.6	2.99	0.065	0.011	0.416
**Time/Visit**	s/V	14.9	16.5	12.5	12.1	15.7	12.3	13.7	14.3	0.03	0.079	0.780	0.386
**Time/Day**	min/d	20.7	14.9	15.9	7.7	17.8	11.8	18.3	11.3	7.57	0.038	0.016	0.381
**Last week of nursery**													
**Visit/Day**	V/d	96.0^b^	155.3^a^	122.8^a^ ^,^ ^b^	93.2^b^	126	108	109	124	3.02	0.315	0.473	0.001
**Time/Visit**	s/V	22.3^p^	16.3^q^	12.7^q^	14.2^q^	19.3	13.4	17.5	15.3	0.04	<0.001	0.046	0.072
**Time/Day**	min/d	33.3^p^	38.3^p^	27.8^p^ ^,^ ^q^	19.4^q^	35.8	23.6	30.6	28.9	4.03	0.007	0.483	0.067

1 RSD: Residual Standard Deviation.

a,b : significant differences (*P*-value < 0.05).

p,q : tendency (*P*-value < 0.10).

V/d: visits per day; s/V: number of visits; min/d: minutes per day.

The sample size for the analysis of feeding behavior in the in the first week were Fast_Fast (8), Fast_Slow (6), Slow_Fast (10), and Slow_Slow (6); in the last week were Fast_Fast (7), Fast_Slow (9), Slow_Fast (8) and Slow_Slow (6).

Data were obtained from each piglet for 24 h during the last 7 d of nursery period.

Data presented as EMMs.

During the last week of the nursery period (51 to 58 d-old) a significant interaction was observed between suckling and nursery growth rate in the variables “visits per day” (*P* = 0.001) and tendency in the interaction “time per visit” (*P* = 0.072) and “time per day” (*P* = 0.067) at the feeder. *P*-values reported below reflect Tukey-adjusted post hoc comparisons for this significant interaction. Fast-slow piglets showed a higher number of visits to the feeder than slow-slow piglets (*P* = 0.018) and fast-fast piglets (*P* = 0.031). The slow-slow piglets tended to spend less time at the feeder per day compared to both the fast-slow groups (*P* = 0.013) and fast-fast piglets (*P* = 0.088). Finally, the fast-fast piglets showed a tendency to spend more time in the feeder per visit than the other groups (*P* < 0.02). The results of the interaction with the feeder are summarized in Table 2.

### Serum and urine metabolites

No significant differences or tendency were found in the interaction between suckling and nursery groups growth rate for the metabolites analyzed in serum and urine. Therefore, the *P*-values shown correspond to the main effects. The serum concentrations of urea, albumin, Zn, and GABA for each group are summarized in Table 3. During the suckling period, serum urea tended to be lower (*P* = 0.087; 95% CI: fast 8.27–18.8 vs. slow 10.3–22; Cohen’s *d *= −0.53), and serum albumin tended to be higher (*P* = 0.079; 95% CI: fast 1.66–2.68 vs. slow 1.54–2.55; Cohen’s *d *= 0.54) in piglets with fast growth than the piglets with slow growth. Serum GABA showed significantly higher levels (*P* = 0.011) in fast-growing piglets during suckling than their counterparts. Finally, serum Zn concentrations were significantly higher in piglets with fast growth during the suckling (*P* = 0.013) and nursery period (*P* < 0.001) than in slow-growth pigs.

**Table 3.** Serum concentrations of urea, albumin, GABA and zinc for the different growth groups during suckling and nursery periods in 58-d-old pigs

Fixed effect	Suckling			Nursery		RSD^1^	*P-*value		
Variables	Units	Fast	Slow	Fast	Slow		Suckling	Nursery	Suckling × Nursery
**Urea**	mg/dL	13.5	16.1	13.6	16.1	4.92	0.087	0.105	0.505
**Albumin**	g/dL	2.17	2.05	2.15	2.07	0.226	0.079	0.254	0.441
**Zinc**	µg/L	764	683	787	661	103.1	0.013	<0.001	0.117
**GABA**	µg/L	65.4	56.9	62.9	59.4	10.22	0.011	0.288	0.561

1 RSD: Residual Standard Deviation.

The sample size for the analysis of difference growth in Fast_Fast (11), Fast_Slow (12), Slow_Fast (11), and Slow_Slow (11).

Outliers were observed in GABA: Fast-Fast (1), Slow-Fast (2); and in Urea: Slow-Slow (1). Outliers correspond to extremely high values

Data presented as EMMs.

Interaction data available in online Supplementary Material.

In urinary metabolites, significant differences were found between piglets with different growth during suckling and nursery periods. Fast-growing piglets during suckling period had lower urea/creatinine (urea/Cr; *P* = 0.030), sulfur/creatinine (sulfur/Cr; *P* = 0.003), KYN/creatinine (KYN/Cr; *P* < 0.001), TRP/creatinine (TRP/Cr; *P* = 0.022) and tended to a lower ratio between KYN/TRP (KTR, *P* = 0.084; 95% CI: fast −0.31 to 6.93 vs. slow 0.916–7.6; Cohen’s *d *= −0.59) concentration in urine compared to slow-growing pigs. Looking at the nursery classification, it was observed that KYN/Cr was lower in fast-growing piglets (*P* = 0.027) and the KTR concentration also tended to be lower compared to slow-growing piglets (*P* = 0.074; 95% CI: fast 0.323–6.36 vs. slow 0.049–8.4; Cohen’s *d *= −0.61). The results of urinary metabolites concentrations among groups are summarized in Table 4.

**Table 4.** Concentration of urea/creatinine, sulfur/creatinine, kynurenine, tryptophan, and kynurenine/tryptophan ratio in urine for the different growth groups during the suckling and nursery periods in 58-d-old pigs

Fixed effect		Suckling		Nursery		RSD^1^	*P-*value		
Variables	Units	Fast	Slow	Fast	Slow		Suckling	Nursery	Suckling × Nursery
**Urea/Creatinine**	mg/dL	7.81	10.46	8.37	9.90	0.495	0.030	0.136	0.587
**Sulfur/Creatinine**	mg/dL	0.89	1.11	0.98	1.03	0.208	0.003	0.434	0.579
**KYN/Creatinine**	ng/mg	17.6	29.1	20.4	26.3	0.039	<0.001	0.027	0.833
**TRP/Creatinine**	ng/mg	575	723	643	655	183.7	0.022	0.845	0.198
**KYN/TRP**	×10^2^	3.31	4.26	3.34	4.22	0.129	0.084	0.074	0.193

1 RSD: Residual Standard Deviation.

KYN: Kynurenine; TRP: Tryptophan. KYN/TRP: Kynurenine/Tryptophan ratio.

The sample size for the urine analysis: Fast_Fast (9). Fast_Slow (12). Slow_Fast (10). and Slow_Slow (11).

Outlier were observed in KIN/creatinine, TRP/Creatinine and KIN/TRP in Fast_Fast (1), Slow_Fast (2) and Slow_Slow (2).

Data presented as EMMs.

Interaction data available in online supplementary material.

### Jejunum gene expression

From the different jejunal genes analyzed, a total of 11 genes showed significant differences or tendencies associated with growing rates groups as is shown in Table 5. Although the FDR-adjusted *P*-values did not reach statistical significance, the nominal *P*-values were still considered as indicative of potential effects. The modest sample size, the limited number of genes analyzed (43), and the fact that these genes were selected as a priori for their primary functional relevance to the hypothesis under investigation support the interpretation of nominal *P*-values despite the lack of FDR-adjusted significance. Regarding the main effects during the suckling period, immune response genes analysis showed that fast-growing piglets exhibited a significant lower mRNA abundance of genes *CXCL2* (*P* = 0.042) and higher mRNA levels of *TLR2* (*P* = 0.025), as well as showing a tendency to lower mRNA abundance of *S1009* (*P* = 0.097; 95% CI: fast −0.544 to 2.380, slow −0.098 to 2.520; Cohen’s *d *= −0.52) and *IL-8* (*P* = 0.051; 95% CI: fast 0.469–1.39, slow 0.627–1.66; Cohen’s *d *= −0.60), and higher mRNA abundance of *NF-κB* (*P* = 0.073; 95% CI: fast 0.803–1.14, slow 0.742–1.10; Cohen’s *d *= 0.54) compared to slow-growth pigs. In addition, it was observed that fast-growing piglets in the suckling period tended to lower mRNA expression of genes involved in protein breakdown (*SH3RF2*; *P* = 0.071; 95% CI: fast −0.0206 to 2.19, slow 0.2091–2.39; Cohen’s *d *= −0.55) and stress enzyme (*HSD11β1*; *P* = 0.054; 95% CI: fast 0.577–1.25, slow 0.757–1.40; Cohen’s *d *= −0.60), compared to slow-growing pigs. Additionally, an interaction between suckling and nursery period was observed in the mRNA gene expression of *NF-κB* (*P* = 0.013) where fast-fast growing piglets showed a higher mRNA abundance compared to slow-fast growing piglets (*P* = 0.019) while the fast-slow and slow-slow growing piglets showed no significant differences compared to the other groups (*P* > 0.1). In addition, significant interactions were observed in *HSPA4*, (*P* = 0.034) between growth in suckling and nursery period, however no differences were observed in pairwise comparisons. A tendency was observed in the interaction between growth in suckling and nursery period in mRNA expression of *IL-8, P* = 0.069; *HNMT*, *P* = 0.065; *GBP1, P* = 0.051; and *IDO1*, *P* = 0.081.

**Table 5.** Relative gene expression in the jejunum tissue of different growth groups during suckling and nursery periods in 58-d-old pigs

Fixed effect		Suckling × Nursery				Suckling		Nursery		RSD^1^	*P-*value			*Adjusted P-value (FDR)*		
		Fast		Slow		Fast	Slow	Fast	Slow		Suckling	Nursery	Suckling × Nursery	Suckling	Nursery	Suckling × Nursery
Function	Genes	Fast	Slow	Fast	Slow											
**Immune response**	***CXCL2***	0.85	0.71	1.01	1.09	0.78	1.05	0.93	0.90	0.483	0.042	0.846	0.174	0.527	0.939	0.857
	***S100A9***	1.09	0.74	1.58	0.85	0.92	1.21	1.34	0.79	0.406	0.097	0.141	0.780	0.564	0.881	0.977
	***TLR2***	1.15	1.28	0.91	0.89	1.21	0.90	1.03	1.08	0.453	0.025	0.696	0.580	0.527	0.881	0.976
	***NF-κB***	1.04^b^	0.91^a,b^	0.89^a^	0.95^a,b^	0.97	0.92	0.96	0.93	0.104	0.074	0.297	0.013	0.527	0.881	0.556
	***HSPA4***	0.99	0.85	0.94	1.07	0.92	1.01	0.97	0.96	0.212	0.184	0.809	0.034	0.657	0.939	0.579
	***IL-8***	1.02	0.84	1.04	1.25	0.93	1.14	1.03	1.04	0.359	0.051	0.900	0.069	0.527	0.941	0.579
	***HNMT***	1.05	0.86	0.98	1.09	0.95	1.04	1.02	0.97	0.262	0.299	0.585	0.065	0.689	0.881	0.579
	***GBP1***	1.19	0.94	0.99	1.41	1.07	1.2	1.09	1.18	0.563	0.412	0.617	0.051	0.689	0.881	0.579
**Digestive enzyme**	***IDO1***	1.52	1.02	1.09	1.25	1.27	1.17	1.30	1.14	0.623	0.563	0.366	0.081	0.807	0.881	0.579
**Protein breakdown**	***SH3RF2***	1.15	1.01	1.26	1.34	1.08	1.30	1.21	1.17	0.416	0.071	0.741	0.377	0.527	0.885	0.957
**Stress enzyme**	***HSD11B1***	0.97	0.86	1.08	1.07	0.92	1.08	1.03	0.97	0.272	0.053	0.444	0.512	0.527	0.881	0.957

1 RSD: Residual Standard Deviation.

^a,b ^Significant differences (P-value < 0.05).

*CXCL2*: Chemokine Ligand 2; *S100A9*: Calcium Binding Protein A9; *TLR2*: Toll Like Receptor 2; *NF-κB*: Nuclear Factor Kappa-betta; *HSPA4*: Heat Shock Protein Family A Member 4; *IL-8*: Interleukin-8; *HNMT*: Histamine N-Methyltransferase; *GBP1*: Guanylate Binding Protein 1; *IDO1*: Indoleamine 2,3-dioxygenase; *SH3RF2*: SH3 Domain Containing Ring Finger 2; *HSD11β1*: Hydroxysteroid (11-beta) dehydrogenase 1.

Outliers were observed in *S100A9* Slow-Fast (1), *IL8* Fast-Fast (1), Slow-Slow (1) and *HSD11β1* Slow-Fast (1)

Data presented as EMMs.

### Correlations

**Figure 2.**
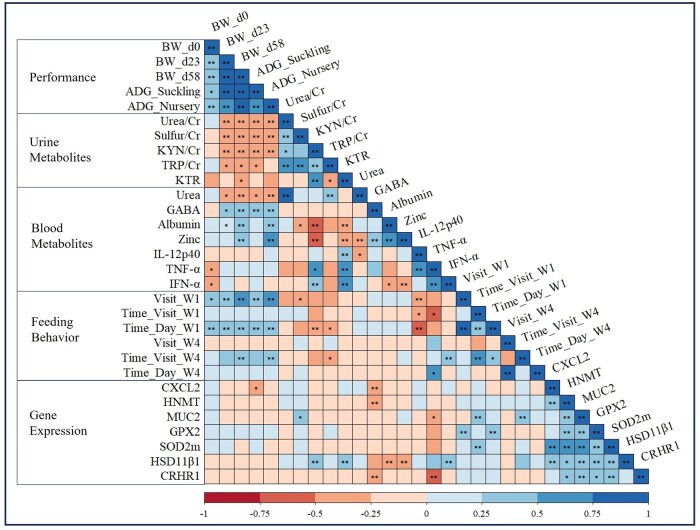
Correlation table of performance parameters, urine and blood metabolites, feeding behavior, and gene expression. ** indicate significance (*P* < 0.05) and * indicate tendency (*P* > 0.05 and <0.1). Figure generated using the software RStudio v4.2.2.

Figure 2 presents the correlation matrix and significance levels for the relationships between performance, blood and urine metabolites, feeding behavior and seven gene expression markers. Significant correlations (*P* < 0.05) were observed between performance and urinary and blood metabolite levels in the animals. Average daily gain during the suckling period showed a positive correlation with GABA in serum (*r* = 0.315), a negative correlation with urea/Cr in urine (*r* = −0.454) KYN/Cr in urine (*r* = −0.444), and sulfur/Cr in urine (*r* = −0.465) and tendency to (*P* < 0.1) a negative correlation with TRP/Cr in urine (*r* = −0.298) and urea in serum (*r* = −0.276). Average daily gain during the nursery period exhibited a significant positive correlation with serum GABA (*r* = 0.330), albumin (*r* = 0.353), and zinc (*r* = 0.560), while showing negative correlations with urea in urine (*r* = −0.413) and blood (*r* = −0.369), KYN/Cr in urine (*r* = −0.440), and sulfur/Cr in urine (*r* = −0.377).

Regarding other correlations between biomarkers or between biomarkers and the expression of different genes, KYN/Cr in urine showed a positive correlation with inflammatory markers, including plasma *TNF-α* (*r* = 0.502; *P* = 0.081) and *IFN-α* (*r* = 0.433; *P* = 0.008), as well as with the jejunal gene expression of the stress-related enzyme *HSD11β1* (*r* = 0.497; *P* = 0.002). Additionally, it showed a significant (*P* < 0.001) negative correlation with nutritional markers as serum albumin (*r* = −0.513; *P* < 0.001) and Zn (*r* = −0.527). Similarly, urine KTR exhibited significative (*P* < 0.05) positive correlations with plasma IL-12p40 (*r* = 0.369), *TNF-α* (*r* = 0.723) and *IFN-α* (*r* = 0.643), and as well as with jejunal *HSD11β1* (*r* = 0.463) and negative correlation with serum albumin (*r* = −0.326) and Zn (*r* = −0.417). Moreover, serum GABA exhibited a significant (*P* < 0.05) negative correlation with jejunal *CXCL2* (*r* = −0.319), *HNMT* (*r* = −0.314), and *CRHR1* (*r* = −0.326), a key marker of the body’s stress response and a significant (*P* < 0.05) negative correlation with serum Zn (*r* = 0.419).

The feeding behavior during the first week showed significant correlations (*P* < 0.05) with performance, metabolites and gene expression related to the intestinal barrier and antioxidant enzymes (*P* < 0.05). Time spent at the feeder per day exhibited significant positive correlation with BW at birth (*r* = 0.393), weaning (*r* = 0.452) and 58 d-old (*r* = 0.498). It was also positively correlated (*P* < 0.05) with ADG during both the suckling (*r* = 0.428) and the nursery (*r* = 0.473) periods, as well as with jejunal *GPX2* gene expression (*r* = 0.404). In contrast, it exhibited a significant negative correlation with KYN/Cr in urine (*r* = −0.406) and plasma IL-12p40 (*r* = −0.511), as well as tendency to a negative correlation with urine KTR (*r* = −237; *P* = 0.079). Time spent per visit to the feeder was significantly positively correlated with jejunal *MUC2* (*r* = 0.386), *SOD2m* (*r* = 0.387) gene expression and showed a tendency to a negative correlation with plasma IL-12p40 (*r* = −0.323; *P* = 0.094) and *TNF-α* (*r* = −0.627; *P* = 0.052). Finally, the number of visits per day to the feeder correlated significantly positively with the ADG during both the suckling (*r* = 0.478) and nursery (*r* = 0.503) periods, jejunal *GPX2* gene expression (*r* = 0.397) and negatively with plasma IL-12p40 (*r* = 0.436). During the final week of the nursery period, time per visit was significantly positively correlated (*P* < 0.05) with BW at day 58 (*r* = 0.392), ADG in nursery (*r* = 0.414) and jejunal *MUC2* gene expression (*r* = 0.42).

## Discussion

The present study has shown that early life experiences, summarized by ADG classifications during the suckling and nursery periods, are crucial in LBW piglets. In effect, a poor start during the suckling period had long-lasting detrimental effects by 58 d-old. The overall nursery phase was also important, as expected considering that weaning is a critical event associated with environmental, nutritional, and social stress. Growth during the suckling period continued to influence piglets at the end of the nursery phase, likely due to a carry-over in growth across periods. However, no statistical interaction was detected between suckling and nursery growth categories, indicating that their effects on the metabolic profile of LBW piglets were largely independent.

### The impact of the suckling period

The main results of this experiment demonstrated that piglets with varying growth rates during suckling showed differences in feeding behavior and metabolism at the end of the nursery period, regardless of their growth rate during this phase. These findings highlighting the influence of early-life conditions, such as environment and feeding status, on subsequent growth and development (Douglas et al., 2014; Paredes et al., 2014; González-Solé et al., 2022).

Animals can adapt their feeding behavior strategies based on social factors, such as their ability to compete. However, this ability also depends on physical limitations, which may increase with BW (Boumans et al., 2015, 2018). In the present study, piglets classified as fast-growth during the suckling period appeared to have an advantage in feeding behavior during the first week post-weaning. These piglets spent more time per day at the feeder shortly after weaning, characterized by more frequent and longer visits, along with a higher ADG during these days compared to slow-growing piglets. Moreover, these feeder interaction variables showed a positive correlation with BW at birth, weaning, and the ADG during the suckling period. In contrast, Bruininx et al. (2001) reported more visits by low BW piglets (6.7 kg) at weaning compared to heavier (9.3 kg) counterparts over the 34 d post-weaning, while Paredes et al. (2014) found no behavioral differences between groups (4.7 vs 7.1 kg) at 9-wk-old. These discrepancies may result from differences in the evaluation period and low-BW classification. Conversely, this study focused on the first week post-weaning, comparing piglets weighing 6.1 vs 4.1 kg at weaning, and specifically included LBW piglets in the growth groups definitions, which likely contributed to the observed differences.

Positive correlations were observed between BW at birth, weaning, and at 58 d-old, as well as with ADG during the suckling and nursery periods. Notably, unintentional birth BW differences, likely inherent to the selection criteria based on ADG were observed, with slow-growth suckling piglets born 97 g lighter than fast-growth counterparts. In addition, slow-growth suckling piglets showed higher excretion of urine urea/Cr and sulfur/Cr, as well as the higher abundance of the gene *SH3RF2* in the jejunum, a gene which may potentially promote the ubiquitination and degradation of target proteins (Wang et al., 2025a). Although measuring *SH3RF2* in muscle would have provided a more direct assessment, the fact that *SH3RF2* is not a canonical muscle E3 ligase suggest that the jejunal findings, combined with the metabolite profiles, may indicate increased catabolic activity 5 wk after weaning. This may result from reduced feed intake and lower lean tissue deposition, disrupting amino acid metabolism. The overall findings are consistent with Romero et al. (2022) who reported that carcasses from LBW piglets had higher fat content and intramuscular fat. However, compensatory neonatal care practices increased carcass leanness in LBW pigs, highlighting the long-term effects of early-life events on protein and lipid metabolism. Therefore, further investigation is warranted including muscular tissue.

Furthermore, LBW piglets exhibit impaired nutrient absorption, compromised immune and underdeveloped gastrointestinal (GI) tract, reflected in lower ADG by the end of the suckling period (Camp Montoro et al., 2020; Villagómez-Estrada et al., 2022). These disadvantageous conditions lead to long-lasting physiological alterations up to 10-wk-old (Michiels et al., 2013; Paredes et al., 2014), as observed in the metabolic effects of poor growth during the suckling period in the present study. Comparable effects on feeding behavior may occur, as in this experiment slow-growing piglets maintain their weaning feeder interaction patterns until 58 d-old. The physiological conditions, combined with behavioral ones, contribute to inadequate feed intake and reduced nutrient flow, leading to inflammation and impaired metabolism (Humphrey et al., 2019). This metabolic disruption in slow-growth pigs is further evidenced by the increased *HSD11β1* gene mRNA abundance in jejunum, which regulates tissue glucocorticoid levels by converting the inactive glucocorticoid cortisone into its biologically active form, cortisol (Stewart, 2003).

The *HSD11β1* gene is positively regulated by pro-inflammatory cytokines such as IFN-γ and TNF-α, and plays a critical role in inflammatory responses (Huang et al., 2020), as also reflected in our results, where its expression positively correlated with KYN/Cr, KTR, and IFN-α. González-Solé et al. (2022) also reported that slow-growing pigs exhibited a higher cortisol-to-cortisone ratio. Glucocorticoids produced locally by *HSD11β1* may induce catabolic effects in skeletal muscle through protein degradation pathways and by suppressing protein synthesis. As a result, lower muscle mass is associated with higher *HSD11β1* expression (Braun and Marks, 2015; Schluessel et al., 2023).

Increased mRNA abundance of jejunal *HSD11β1* gene in slow-growth pigs, along with the reduced serum albumin and Zn levels, indicating prolonged dysregulation of metabolic and inflammatory pathways associated with impaired health, nutritional deficiency, and chronic stress (Cieślik et al., 2007; Soeters et al., 2019; Huang et al., 2020). These negative cycles during suckling may impose a greater physiological burden until slaughter, delaying market weight even when strategies such as later weaning or improved early post-weaning diets have been implemented(Wolter and Ellis, 2001; Cabrera et al., 2010; Huting et al., 2019).

Behavioral differences linked to early growth may reflect inherent variations in social interactions related to the metabolic adaptations. Although research in this area is limited in pigs, studies in other species may provide insights into metabolic indicators linked to behavioral or mental health conditions, such as the TRP metabolism, which occurs primarily through the 5-HT and KYN, pathways related to psychological processes and mediated by inflammatory response (Zheng et al., 2010; Li et al., 2022; Correia and Vale, 2024). Fast-growing suckling piglets showed higher mRNA abundance *NF-kB* and *TLR2* gene expression in the jejunum, which may indicate increased inflammatory signaling. However, the lack of overexpression of proinflammatory jejunal genes *CXCL2*, *S100A9*, and *IL-8* (Russo et al., 2014; Cerón et al., 2023) suggests a reduced need for intense inflammatory responses, indicating a more balanced or efficient GI immune state.

Moreover, *TLR2* also mediates microbiota-induced 5-HT production in the gut (Wang et al., 2019), and its lower mRNA abundance in slow-growing suckling piglets may reflect a metabolic–microbial imbalance that reduces 5-HT, a neurotransmitter involved in mood, stress, appetite, memory, and GI function (Correia and Vale, 2024). In addition, slow-growth piglets during the suckling period showed higher urinary KYN/Cr and KTR levels compared to their fast-growing counterparts. Although cytokine levels did not differ, urine KYN/Cr showed a positive correlation with plasma IFNα, while urine KTR correlated positively with plasma IL-12p40 and TNFα, elucidating the link between urine KTR and the inflammatory response, suggesting the activation of TRP metabolism to KYN by tryptophan-2,3-dioxygenase (TDO), or indoleamine 2,3-dioxygenase (IDO) (Li et al., 2022). It is worth noting that lower albumin in slow-growth piglets during suckling could theoretically increase free TRP and KTR. However, this effect depends on the underlying cause of albumin reduction; if albumin decline reflects poor health and reduced synthesis, the overall TRP pool may also decrease. Furthermore, jejunal expression levels of *CXCL2, S100A9* and *IL-8* may suggest the activation of TRP metabolism to KYN by TDO/IDO (Li et al., 2022).

Studies in rats and humans have reported that higher urinary TRP and KYN levels, and lower plasma GABA have been associated with depressive disorders involving hypothalamic–pituitary–adrenal axis hyperactivity, raising cortisol levels (Zheng et al., 2010; Liwinski et al., 2023). Accordingly, our results showed that slow-growing suckling piglets have reduced serum GABA levels compared to fast-growing piglets. Additionally, there was a positive correlation between urine KYN/Cr and KTR with expression of the jejunal *HSD11β1* gene, suggesting a link between dysregulated KYN metabolism and corticosteroid activity, and a negative correlation between serum GABA levels and expression of the jejunal *CRHR1*, *CXCL2*, and *HNMT* gene, indicators related to inflammation, depression, and sleeping disorder (Koriem and Tharwat, 2023; Liwinski et al., 2023; Ma et al., 2024). These findings align with previous research that showed GABA supplementation in pigs reduces adrenocorticotropic hormone and cortisol levels, decreases negative social behaviors, and increases feeding time (Li et al., 2015), which supports its beneficial role in social behavior, feeding activity, and stress regulation.

### The impact of the nursery period

Piglets exhibiting varying growth rates after weaning were associated with subsequent differences at 58 d-old. Weaning is the most critical nursery event, and a successful transition is crucial for future growth. Weaning BW showed a strong positive correlation with BW at 58 d-old and ADG during the nursery period, which is a well-established predictor of growth not only during the nursery phase but also up to slaughter (López-Vergé et al., 2018).

Fast-growing nursery piglets exhibited more visits and longer daily interaction times with the feeder during the first week postweaning. Furthermore, they showed a higher ADG during this week, and both ADG during the nursery period and BW on day 58 showed positive correlations with these feeding behavior variables. This is particularly relevant because these results are retrospective and no interaction between growth during suckling and nursery was observed, which may serve as a predictor of growth performances, similarly to weaning BW. Indeed, Fabà et al. (2024) observed that the feed intake during the first three days of postweaning is an independent factor in addition to BW at weaning, for predicting future growth, and is positively associated with the number of visits and time spent at the feeder. Piglets with higher feeding intake exhibited greater growth and spent more time per day at the feeder and made more frequent visits (Fabà et al., 2024), consistent with the results of the current study.

During the first week of the nursery period, total daily feeder time positively correlated with antioxidant enzyme jejunal *GPX2* gene expression, while the time spent per feeder visit positively correlated with barrier-related jejunal gene *MUC2 and SOD2m* expression, suggesting an improvement in the future development of GI health (Villagómez-Estrada et al., 2022; Wang et al., 2025b). This supports the idea that in our study, piglets spending more time at the feeder consume more feed. Additionally, piglets with higher feed intake during the first three days of the nursery period showed an enhanced metabolic and intestinal physiological profile (Fabà et al., 2024), reflecting a faster adaptation to the new environment and learning feeder interaction skills, both critical for their development. Moreover, as observed during the suckling period, piglets with slow growth during the nursery period showed evidence of protein catabolism and nutritional limitations, as supported by lower zinc levels in blood and positive correlations between ADG in the nursery period and plasma levels of urine urea/Cr, sulfur/Cr, serum urea, albumin, and zinc. Furthermore, higher urinary KYN/Cr and KTR levels, and positive correlation between ADG in the nursery period with GABA in serum, suggest activation of the TDO/IDO pathways, linking slow-growing piglets to a depressive-like state and social behavioral challenges.

Furthermore, at the end of the nursery period, it becomes evident that growth in both the suckling and nursery period significantly influences pigs’ behavior. Positive correlations were observed between time per visit during the last week of the nursery and BW at 58 d-old, as well as ADG during the nursery period. Moreover, slow-slow animals made a similar number of daily visits to the feeder as fast-fast animals; however, they spent less time interacting with the feeder and had shorter visit durations. Similar patterns were reported in growing pigs by Carcò et al. (2018), suggesting behavior at this point resembles that of larger pigs. An increase in meal frequency patterns and a decrease in meal duration, as well as a reduction in meal size, are indicative of a competitive feeding environment and imply high levels of displacement (Boumans et al., 2015). Therefore, these results may suggest that slow-slow animals are more susceptible to interruptions and exhibit lower competitiveness. Although individual feeding intake per visit was not measured, fast-growing piglets likely consumed more per visit during the nursery period, supported by their higher ADG in the last week. This suggests slow-growing piglets may experience earlier satiety, reinforcing their nutritional disadvantage and potential social stress.

## Conclusion

Early-life experiences in piglets, particularly during the suckling period, have a significant impact on late performance, as reflected in the physiological and behavioral differences observed at the end of the nursery period. Slow growing piglets exhibit activation of metabolic pathways related to inflammation, protein catabolism, stress, and depression suggesting that early growth restrictions may trigger long-lasting physiological alterations. Although the study has certain limitations, such as the absence of direct muscle sampling to confirm catabolic pathways and a sample size that could have been expanded to increase statistical power, the findings underscore the need for deeper investigation of specific physiological variables. Importantly the results clarify that the suckling and nursery periods exert independent effects on the growth, physiological status, and behavior. This understanding opens new research opportunities focused on identifying physiological and behavioral markers, including those associated with depressive-like states in piglets with poor early growth, which could ultimately support the development of novel intervention strategies.

## Supplementary Material

skaf437_Supplementary_Data

## Abbreviations:

5-HT: serotonin;
ADG: average daily gain;
ANOVA: analysis of variance;
*CRHR1*,: *corticotropin releasing hormone receptor 1;*
*CXCL2*: *chemokine ligand 2;*
d-old: day old;
EMMs: estimated marginal means;
GABA: gamma-aminobutyric acid;
*GBP1*: *guanylate binding protein 1;*
GI: gastrointestinal;
*GPX2*: *glutathione peroxidase;*
*HNMT*: *histamine N-methyltransferase;*
*HSD11β1*: *hydroxysteroid (11-beta) dehydrogenase 1;*
*HSPA4*: *heat shock protein family A member 4;*
*IDO1*: *indoleamine 2,3-dioxygenase 1;*
IFN-α: interferon-alpha;
IGF-I: insulin-like growth factor;
IL-12p40: interleukin-12 subunit p40;
*IL-8*: *interleukin 8;*
KTR: kynurenine/tryptophan ratio;
KYN: kynurenine;
KYN/Cr: kynurenine/creatinine ratio;
LBW: low birth weight;
min/d: minutes per day;
*MUC2*: *mucin 2;*
*NF-κB*: *nuclear factor kappa-betta;*
RSD: residual standard deviation;
s/V: seconds per visit;
*S100A9*: *S100 calcium binding protein A9;*
*SH3RF2*: *E3 ubiquitin-protein ligase;*
*SOD2m*: *superoxide dismutase;*
Sulfur/Cr: sulfur/creatinine ratio;
*TLR2*: *Toll-like receptor 2;*
TNF-α: tumor necrosis factor alpha;
TRP: tryptophan;
TRP/Cr: tryptophan/creatinine ratio;
Urea/Cr: urea/creatinine ratio;
V/d: visits per day
