# Supplementary material for: Early-life stages impact later feeding behavior and physiology in light-born piglets
Source: J Anim Sci. 2025 Dec 18;104:skaf437. doi: 10.1093/jas/skaf437 (PMC12863952; doi:10.1093/jas/skaf437)
Supplement: skaf437_Supplementary_Data [file skaf437_supplementary_data.zip › 03-Jan-2026_011704_Supplementary_material_Huenul_et_al.,_2025.docx]

**Supplementary material**

**1.** Estimated marginal means (Emmean) average daily gain (ADG) per week of subset selected for the study.

| **Groups** | | **Average Daily Gain, g/d** | | | | | |
| --- | --- | --- | --- | --- | --- | --- | --- |
|  |  | Day 0-7 | Day 23-30 | Day 30-37 | Day 37-44 | Day 44-51 | Day 51-58 |
| *Entire group^2^* | | 106 | 215 | 160 | 207 | 288 | 336 |
| **Lactation** | **Nursery** |  |  |  |  |  |  |
| Fast | Fast | 146 | 277 | 225 | 225 | 368 | 422 |
|  | Slow | 136 | 183 | 125 | 125 | 299 | 350 |
| Slow | Fast | 86 | 234 | 185 | 185 | 303 | 273 |
|  | Slow | 76 | 162 | 105 | 105 | 183 | 225 |
| **Suckling** | |  |  |  |  |  |  |
| Fast | | 141 | 230 | 175 | 175 | 334 | 386 |
| Slow | | 81 | 198 | 145 | 145 | 243 | 249 |
| **Nursery** | |  |  |  |  |  |  |
| Fast | | 116 | 255 | 205 | 205 | 335 | 348 |
| Slow | | 106 | 173 | 115 | 115 | 241 | 288 |
| **RSD^1^** | | 26.8 | 40.9 | 62.7 | 77.4 | 67.0 | 74.1 |
| ***P-*value** | |  |  |  |  |  |  |
| Suckling | | <0.001 | 0.053 | 0.121 | 0.001 | <0.001 | <0.001 |
| Nursery | | 0.274 | <0.001 | <0.001 | 0.022 | <0.001 | 0.019 |
| Suckling x Nursery | | 0.989 | 0.449 | 0.597 | 0.356 | 0.209 | 0.635 |
| ^1^: RSD: Residual Standard Deviation.  *^2^:* Mean value for all animals used in the experiment and timepoint. | | | | | | | |

- 1. ***Table 4:*** Serum concentrations of urea, albumin, GABA and zinc for the different growth groups during lactation and nursery periods in 58-day-old pigs.

| 58 d old pigs | | **Urea** | **Alb**u**min** | **Z**i**n**c | **GABA** |
| --- | --- | --- | --- | --- | --- |
|  |  | **mg/dl** | **g/dl** | **µg /L** | **µg/L** |
| **Suckling** | **Nursery** |  |  |  |  |
| Fast | Fast | 12.8 | 2.18 | 802 | 68.1 |
|  | Slow | 14.4 | 2.16 | 726 | 62.8 |
| Slow | Fast | 14.2 | 2.11 | 771 | 57.7 |
|  | Slow | 17.9 | 1.98 | 595 | 56.1 |
| **RSD**^1^ | | 4.92 | 0.226 | 103 | 10.22 |
| Suckling x Nursery | | 0.505 | 0.441 | 0.117 | 0.561 |
| ^1^RSD: Residual Standard Deviation.  The sample size for the analysis of difference growth in Fast_Fast (11), Fast_Slow (12), Slow_Fast (11), and Slow_Slow (11).  Outliers were observed in GABA: Lactation Fast-Fast (1), Slow-Fast (2); and in Urea: Lactation Slow-Slow (1). Outliers correspond to extremely high values  Data presented as estimated marginal means (Emmean). | | | | | |

- 1. **Table 5:** Concentration of urea/ creatinine, sulphur/ creatinine, kynurenine, tryptophan, and kynurenine / tryptophan ratio in urine for the different growth groups during the lactation and nursery periods in 58-day-old pigs.

| 58 day old pigs | | **Urea/** **Creatinin**e | **Sulphur/** **Creatinin**e | **KYN/ Creatinin**e | **TRP/ Creatinin**e | **KYN/TRP ratio** |
| --- | --- | --- | --- | --- | --- | --- |
|  |  | **mg/dl** | mg/dl | ng/mg | ng/mg |  |
| **Suckling** | **Nursery** |  |  |  |  |  |
| Fast | Fast | 7.49 | 0.89 | 16.4 | 528 | 3.45 |
|  | Slow | 8.13 | 0.91 | 18.9 | 622 | 3.17 |
| Slow | Fast | 9.26 | 1.06 | 24.5 | 758 | 3.23 |
|  | Slow | 11.67 | 1.15 | 33.7 | 687 | 5.28 |
| **RSD**^1^ | | 0.495 | 0.208 | 0.039 | 183.7 | 0.129 |
| Suckling x Nursery | | 0.587 | 0.579 | 0.833 | 0.198 | 0.193 |
| ^1^RSD: Residual Standard Deviation.  KYN: Kynurenine; TRP: Tryptophan. KYN/TRP: Kynurenine / Tryptophan ratio.  KYN/TRP is presented as Emmean x10^2^  The sample size for the urine analysis: Fast_Fast (9). Fast_Slow (12). Slow_Fast (10). and Slow_Slow (11).  Outlier were observed in KIN/creatinine, TRP/Creatinine and KIN/TRP in Fast_Fast (1), Slow_Fast (2) and Slow_Slow (2).  Data presented as estimated marginal means (Emmean). | | | | | | |
